# Supplementary material for: Megadomains and superloops form dynamically but are dispensable for X-chromosome inactivation and gene escape
Source: Nat Commun. 2018 Nov 27;9:5004. doi: 10.1038/s41467-018-07446-w (PMC6258728; doi:10.1038/s41467-018-07446-w)
Supplement: Supplementary file 1 — Supplementary Information [file 41467_2018_7446_MOESM1_ESM.docx]

**Megadomains and superloops form dynamically but are dispensable for X-chromosome inactivation and gene escape**

**Froberg et al.,**

**SUPPLEMENTARY FIGURES**


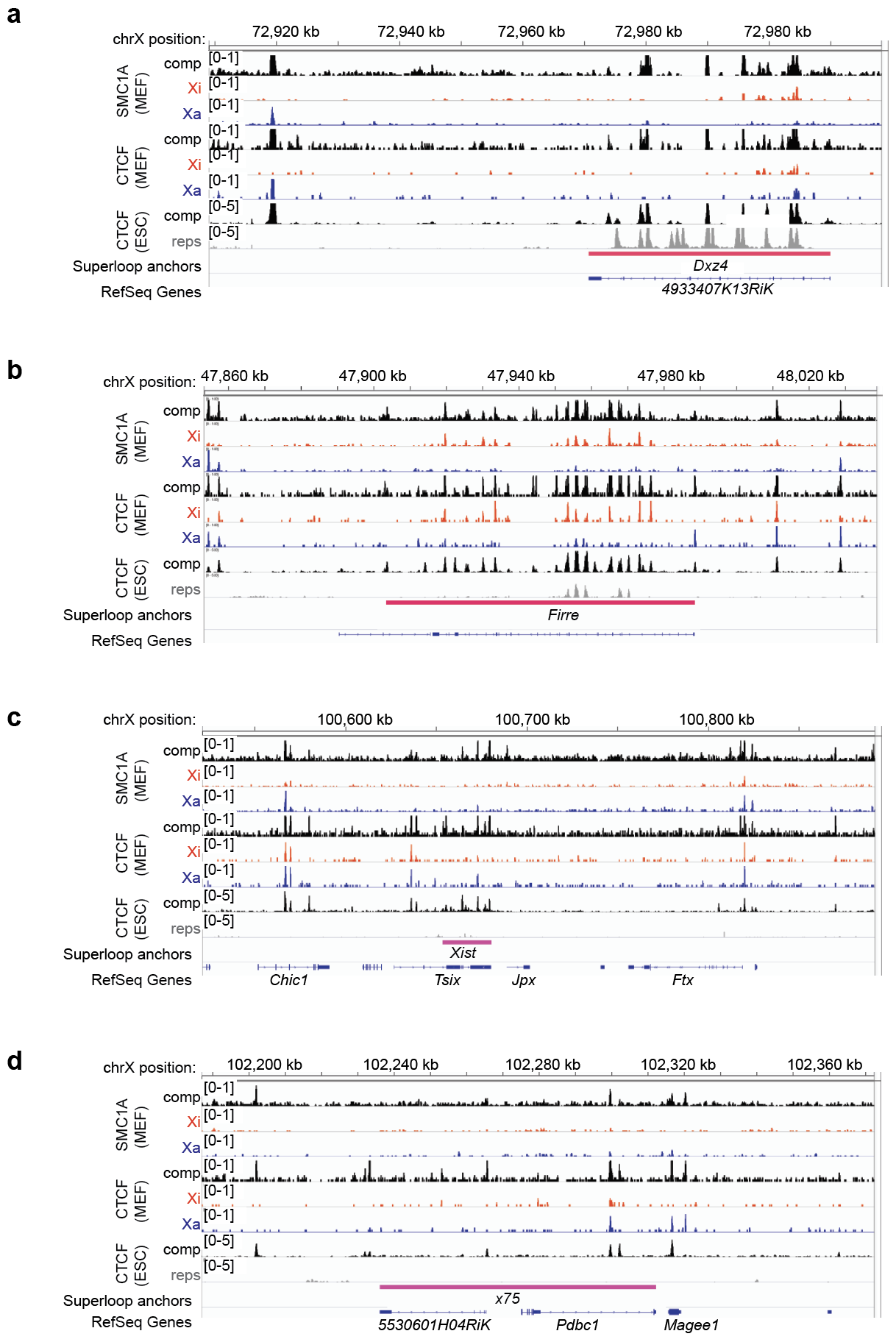


**Supplementary Figure 1: Superloop anchors are tandem repeats that bind CTCF and SMC1A on the mouse Xi.**

SMC1A and CTCF ChIP-seq coverage across the superloop anchors in mouse (**a**) *Dxz4*, (**b**) *Firre*, (**c**) The X-Inactivation Center, and (**d**) *x75*. From top to bottom within a panel: SMC1A ChIP-seq in MEF, black=comp (all unique reads including neutral, cas, and mus), blue=Xa, red=Xi, CTCF ChIP-seq in MEF (same color scheme), CTCF ChIP-seq in mESCs, black=comp, grey=repetitive alignments.


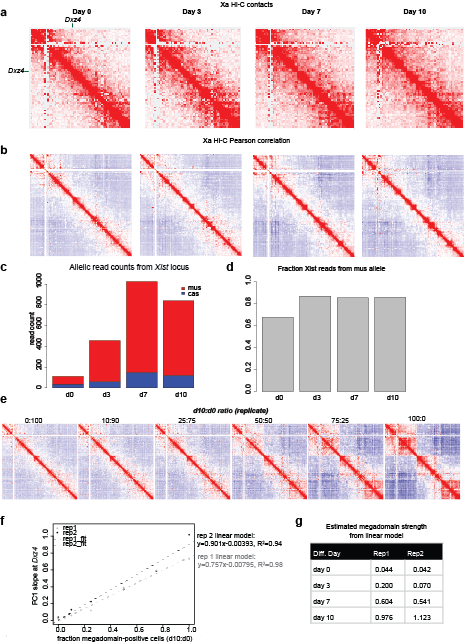


**Supplementary Figure 2: Megadomains are not present on the Xa and allelic RNA-seq shows Xist upregulation is highly skewed to Xi.**

**a** KR-normalized Hi-C matrices on future Xa (cas) on days 0, 3, 7,10 of differentiation (2.5 Mb resolution). **b** Pearson correlation of Hi-C matrix on future Xa (cas) on days 0, 3, 7, 10 of differentiation (1Mb resolution). **c** Numbers of Xist reads expressed from the mus (red) or cas (blue) during differentiation. **d** Fraction of allelic Xist reads expressed from X^mus^ (carrying the *Tsix^TST^* allele) during differentiation, in two biological replicates. **e** Replicate mixing experiment. Pearson correlation of Hi-C matrices at 1 Mb (bottom) for the Xi from data sets generated by mixing an indicated ratio of day 0 (d0, megadomain-negative) and day 10 (d10, megadomain-positive) datasets. This mixing experiment uses data from replicate 1 of the timecourse experiments. **f** Slope of the PC1 score curve at *Dxz4* for Hi-Cs generated with varying ratios of d10:d0 reads. Grey: mixing experiment performed with replicate 1 of the timecourse Hi-Cs; black: mixing experiment performed with replicate 2 of the timecourse Hi-Cs. Dashed lines represent linear best fits; linear model (slope ~ fraction megadomain positive) parameters are included. **g** Estimated megadomain strength for each day in each replicate from the linear models generated in Fig. S2f.

**
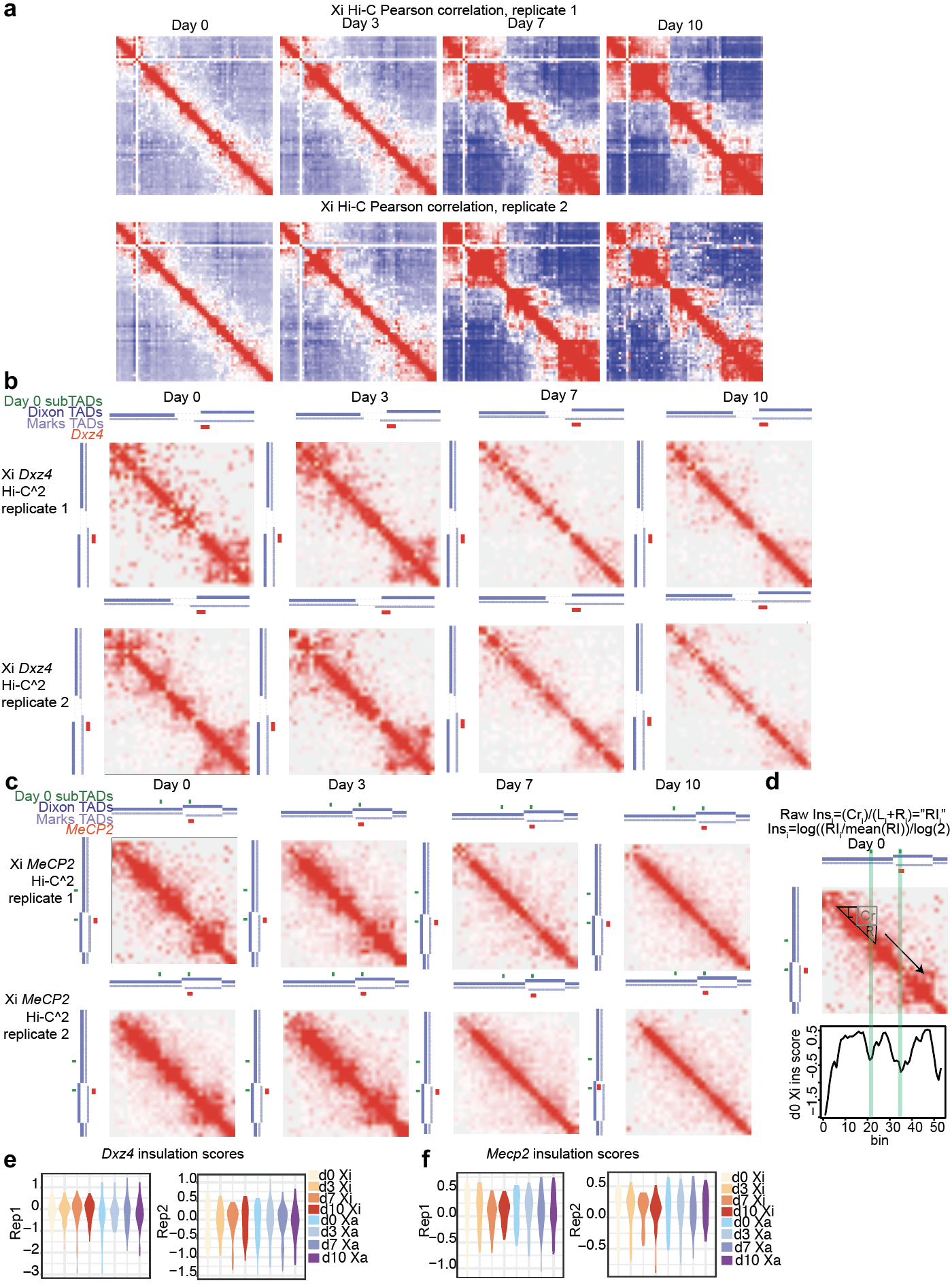
**

**Supplementary Figure 3: Biological replicates of the time course Hi-C and Hi-C^2 experiments.**

**a** Pearson correlation matrices at 2.5 Mb resolution for the Xi (right) days 0, 3, 7, 10 of differentiation in two biological replicate Hi-C experiments. **b** Hi-C^2 contact maps around *Dxz4* (mm9 coordinates chrX:71,832,976-73,511,687) and on the Xi on days 0, 3, 7, 10 of differentiation (50 kb resolution) in two biological replicates. **c** Hi-C^2 contact maps around *Mecp2* (mm9 coordinates chrX:70,370,161-71,832,975) and on the future Xi on days 0, 3, 7, 10 of differentiation (50 kb resolution) in two biological replicates. In **b** and **c**, Green bars indicate positions of domain borders determined from 25 kb d0 comp Hi-C^2 matrices; dark blue track shows Dixon et al. TAD calls in mESCs, light blue track shows Marks et al. TAD calls in mESCs, red bars indicate positions of either *Dxz4* or *Mecp2.* **(d)** Conceptual diagram of insulation score. Top: formula for calculating insulation score at region i. R_i_ refers to the sum of interactions within the region to the right of i, L_i_ refers to the sum of interactions within the region to the left of i, and Cr_i_ represents the sum of interactions that “cross over” i. Middle: diagram of the window used to calculate insulation score for an example (non-border) i in the day 0 Xi *Mecp2* contact map. Bottom: plot of insulation score across the day 0 Xi *Mecp2* region. The shaded green bars indicate the positions of the borders of the Mecp2 sub-TADs across all diagrams. **e, f** Violin plots showing the distributions of insulation scores across the *Dxz4* region (**e**) and *Mecp2* region **(f)** in both biological replicates. Note: to generate violin plots and evaluate the significance of differences in variance between timepoints we excluded the 6 bins on each edge of the Hi-C^2 region because the regions needed to calculate insulation score fall partly outside the Hi-C^2 region and have far lower read counts than sequences targeted by the capture probes.


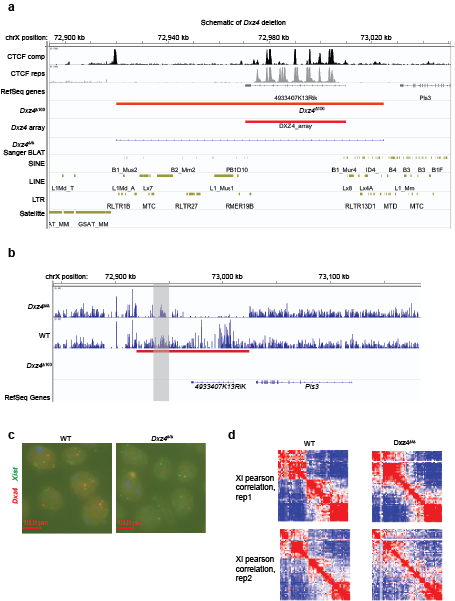


**Supplementary Figure 4: Validation of *Dxz4* deletion and replicate Hi-C.**

**a** Scheme of the *Dxz4* deletion. The large red bar shows the region deleted in this study; the smaller red bar shows the coordinates of the *Dxz4* tandem repeat array. Interspersed repeats are shown in gold, and CTCF ChIP-seq in mESCs is shown in black for all unique reads and grey for all repetitive reads. The “∆Dxz4 Sanger BLAT” track is the BLAT result for Sanger sequencing result from the PCR product generated with primers flanking the deleted region. **b** Hi-C coverage over the *Dxz4* deletion region for *Dxz4*^∆/∆^ clone E5 (top) and WT (bottom). The near-absence of reads in *Dxz4*^∆/∆^ over the deletion implies a biallelic deletion. Note: we observed reads in *Dxz4*^∆/∆^ within the left end of the deletion (grey box), roughly chrX:72,940,913-72,948,483. BLAT analysis of this region indicates that it is repeated several times elsewhere in the genome (data not shown), thus reads within this region are to be expected even in *Dxz4*^∆/∆^.­ **c** DNA FISH in WT (left) and *Dxz4*^∆/∆^ (right) using either *Dxz4* (red) or *Xist* (green) probes. **d** Pearson correlation matrices at 2.5 Mb resolution for the wild-type (left) and *Dxz4*^∆/∆^ Xi (right) in two biological replicate Hi-C experiments. Note: the WT control Hi-C in replicate 2 in this figure also serves as the replicate 2 day 10 timepoint in FigS3a, thus this Pearson correlation matrix is present in both FigS3a and this figure.

**
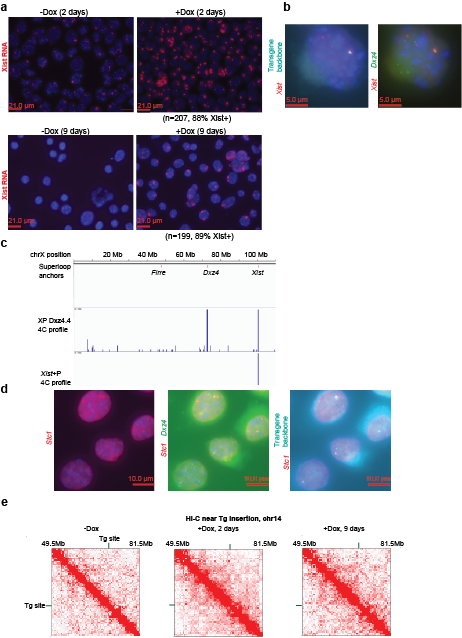
Supplementary Figure 5: Generation of a *Xist+Dxz4* transgene.**

**a** Xist RNA FISH in *Xist+Dxz4* Tg cells (clone XPDxz4.4) +/-Dox after either 2 days of induction (top) or 9 days of induction (bottom panels). The total number of cells and percentage of Xist-positive cells are listed. **b** Co-localization between transgenic *Xist* and *Dxz4*. Left, DNA FISH for the transgene’s P1 backbone (cyan)+*Xist* (red). Right: DNA FISH for *Xist* (red) and *Dxz4* (green). **c** 4C contact profiles in XPDxz4.4 or a separate *Xist*-only transgenic cell line (XY X+P) on chrX from a viewpoint within the backbone of the *Xist* construction. The positions of *Firre, Dxz4* and *Xist* are indicated on the X-chromosome. **d** Co-localization between *Xist* and *Dxz4* and the candidate insertion region (*Stc1*, chr14) obtained from 4C. Left: DNA FISH for *Stc1* (red). Middle: overlap between DNA FISH for *Stc1* (red) and *Dxz4* (green). Right: overlap between DNA FISH for *Stc1* (red) and the Xist transgene’s P1 backbone (cyan). **e** Contact maps in the vicinity of the transgene insertion site on chr14 (500kb resolution).

**
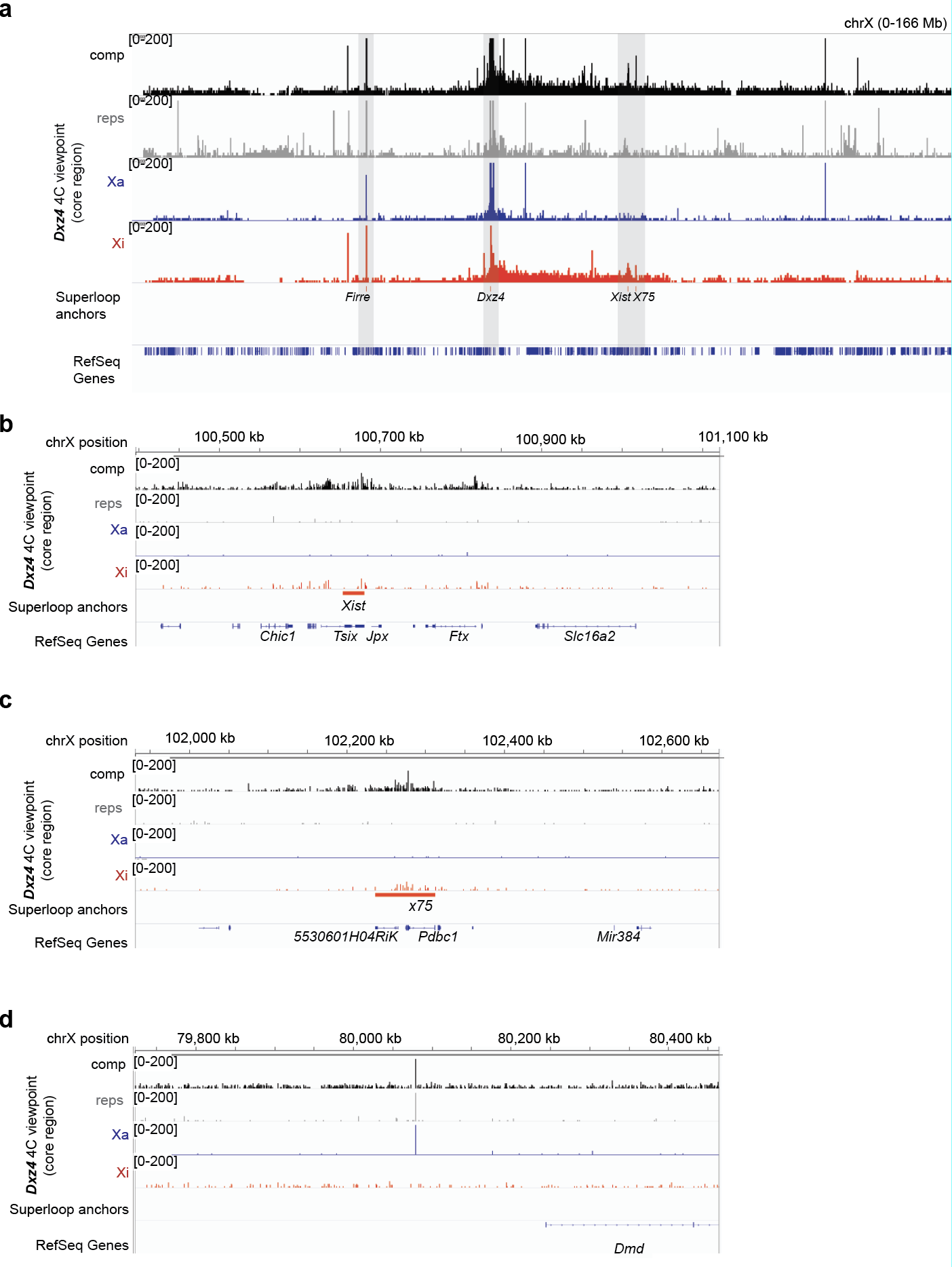
**

**Supplementary Figure 6: A conserved network of superloop interactions involving *Dxz4* on the mouse Xi.**

**a** 4C coverage from the *Dxz4* viewpoint over the whole X in mus Xi MEFs. Black=comp, grey=reps, blue=Xa, red=Xi. The positions of putative superloop anchors *Firre, Dxz4, Xist* and *x75* are shown. **b** 4C coverage from the *Dxz4* viewpoint over the *Xist* region. **c** 4C coverage from the *Dxz4* viewpoint over the region homologous to human x75 in mouse. **d** 4C coverage from the *Dxz4* viepoint over a putative singleton artifact near *Dmd*. Many of the other large peaks on the X or elsewhere are “singletons”, amplified sequences likely due to mispriming, this is an example of a singleton.

**
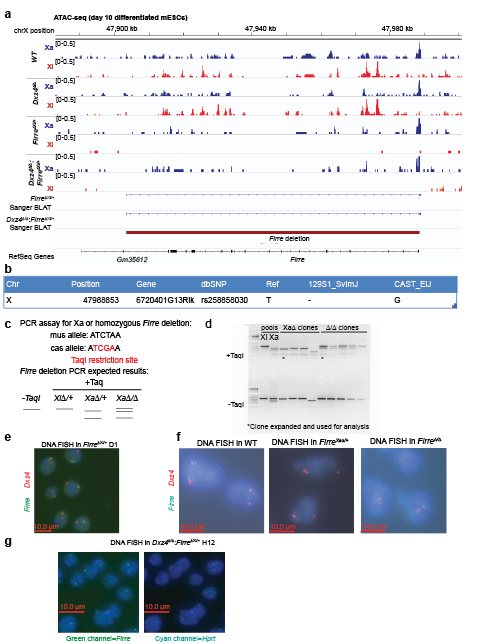
**

**Supplementary Figure 7: Validation of *Firre* deletions.**

**a** Allele-specific ATAC-seq coverage over *Firre*. The coordinates of the *Firre* deletion are shown by the dark red bar, and the two “Sanger BLAT” tracks represent the coordinates of the BLAT alignment of the PCR products flanking *Firre* generated from *Firre*^Xi∆/+^ clone D1 and *Dxz4^∆/∆^:Firre^Xi∆/+^* clone H12. **b** SNP information from the Mouse Genomes Project[^57^](#_ENREF_57) for the SNP that falls within the *Firre* deletion amplicon. **c** Scheme of restriction assay to determine which allele of *Firre* is deleted. **d** Results of restriction assay in Xa- and Xi- *Firre* targeted pools and *Firre*^Xa∆/+^ and *Firre*^∆/∆^ candidate clones. **e** DNA FISH in *Firre*^Xi∆/+^ clone D1 for *Dxz4* (red), and *Firre* (green). **f** DNA FISH for *Dxz4* (red) and *Firre* (cyan) in WT, *Firre*^Xa∆/+^ and *Firre*^∆/∆^ clones. In *Firre*^Xi∆/+^ and *Firre*^Xa∆/+^ most cells exhibit one *Firre* spot but two *Dxz4* spots, consistent with a heterozygous *Firre* deletion, whereas *Firre*^∆/∆^ does not exhibit Firre spots, consistent with a homozygous *Firre* deletion. **g** DNA FISH in *Dxz4*^∆/∆^:*Firre*^Xi∆/+^. Left, *Firre* (green). Right, X-linked gene *Hprt* (Cyan). Most cells exhibit one *Firre* spot but two *Hprt* spots, consistent with a heterozygous *Firre* deletion.

**
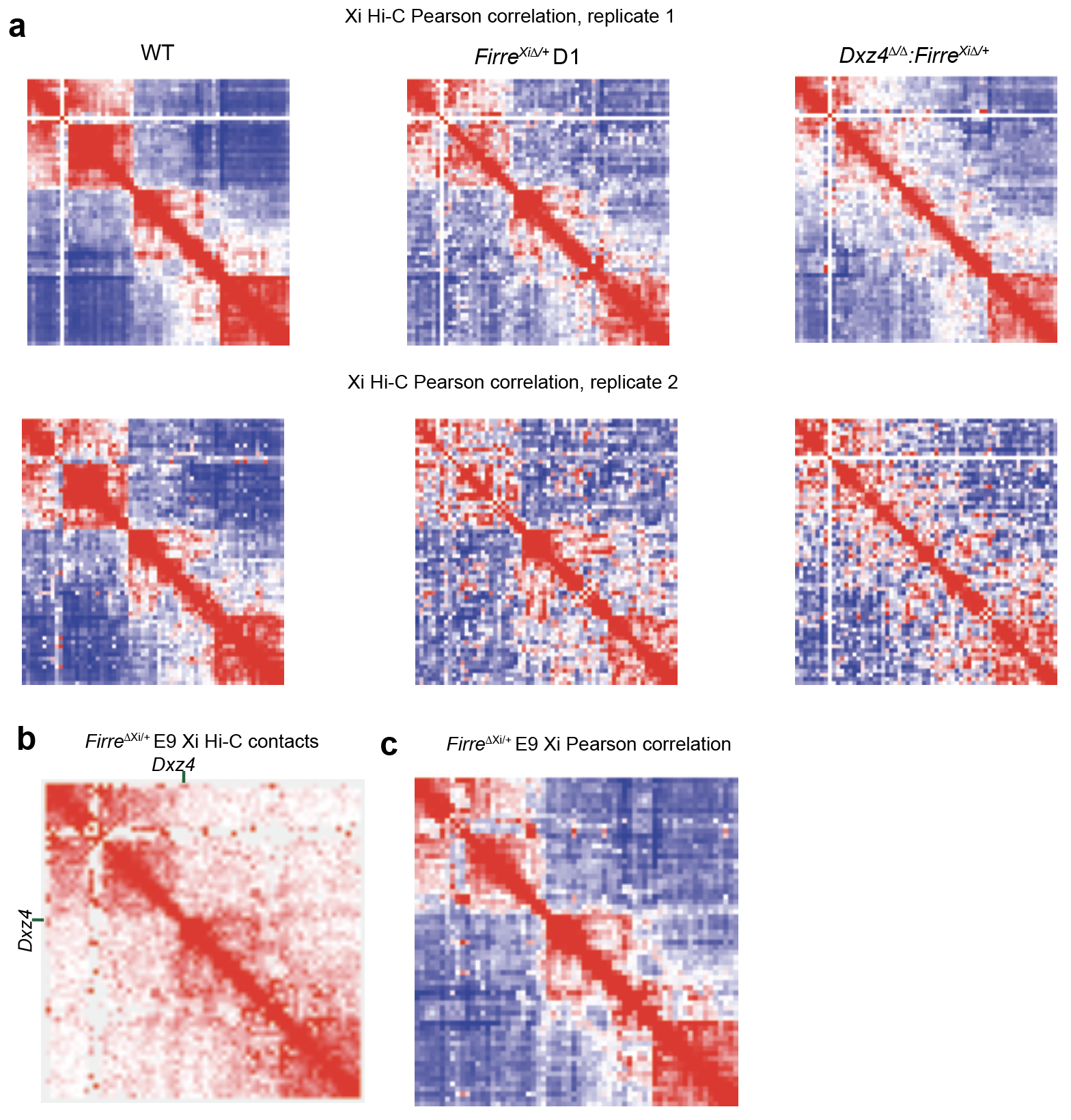
**

**Supplementary Figure 8: Replicate Hi-Cs in *Firre* deletions.**

**a** Pearson correlation matrices at 2.5 Mb resolution for the wild-type (left) and *Firre^Xi^*^∆/+^ (middle, clone D1) and *Dxz4*^∆/∆^:*Firre*^Xi∆/+^ Xi (right) in two biological replicate Hi-C experiments. Note: the WT control Hi-C in replicates 1 and 2 in this figure also serve as the replicates 1 and 2 day 10 timepoints in Fig. S3a, thus these Pearson correlation matrices are present in both FigS2a and the current figure. **b** KR-normalized Hi-C matrix for the independently derived *Firre*^Xi∆/+^ clone (E9) at 2.5 Mb resolution. **c** Pearson correlation matrix for the independently derived *Firre*^Xi∆/+^ clone (E9) at 2.5 Mb resolution.

**
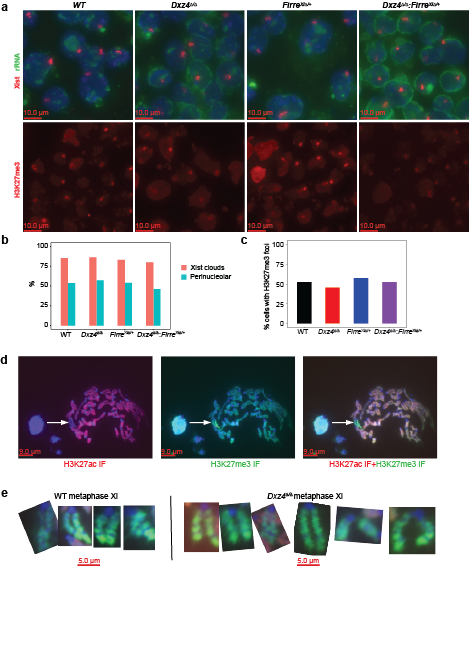
Supplementary Figure 9: Further analysis of Xist localization and H3K27me3 distribution in *Dxz4* and *Firre* deletions.**

**a** Xist RNA FISH (red) combined with rRNA FISH (green) (top), H3K27me3 IF (red, bottom) in WT and *Dxz4*^∆/∆^ *Firre*^Xi∆/+^ and *Dxz4*^∆/∆^:*Firre*^Xi∆/+^ cells on day 10. **b** Fraction of cells with Xist clouds (red) and fraction of Xist clouds in the perinucleolar space (cyan) on day 10. **c** Fraction of cells with an H3K27me3 focus on day 10. **d** Immunoflourescence for H3K27ac (red, left) or H3K27me3 (green, middle) on metaphase spreads from WT cells after 10 days of differentiation. The Xi is readily detectable as the one chromosome in the spread depleted of H3K27ac and enriched in H3K27me3 (merge, right). **e** Coating of H3K27me3 on several metaphase inactive Xs in WT (left) or *Dxz4*^∆/∆^ (right) on day 10.

**
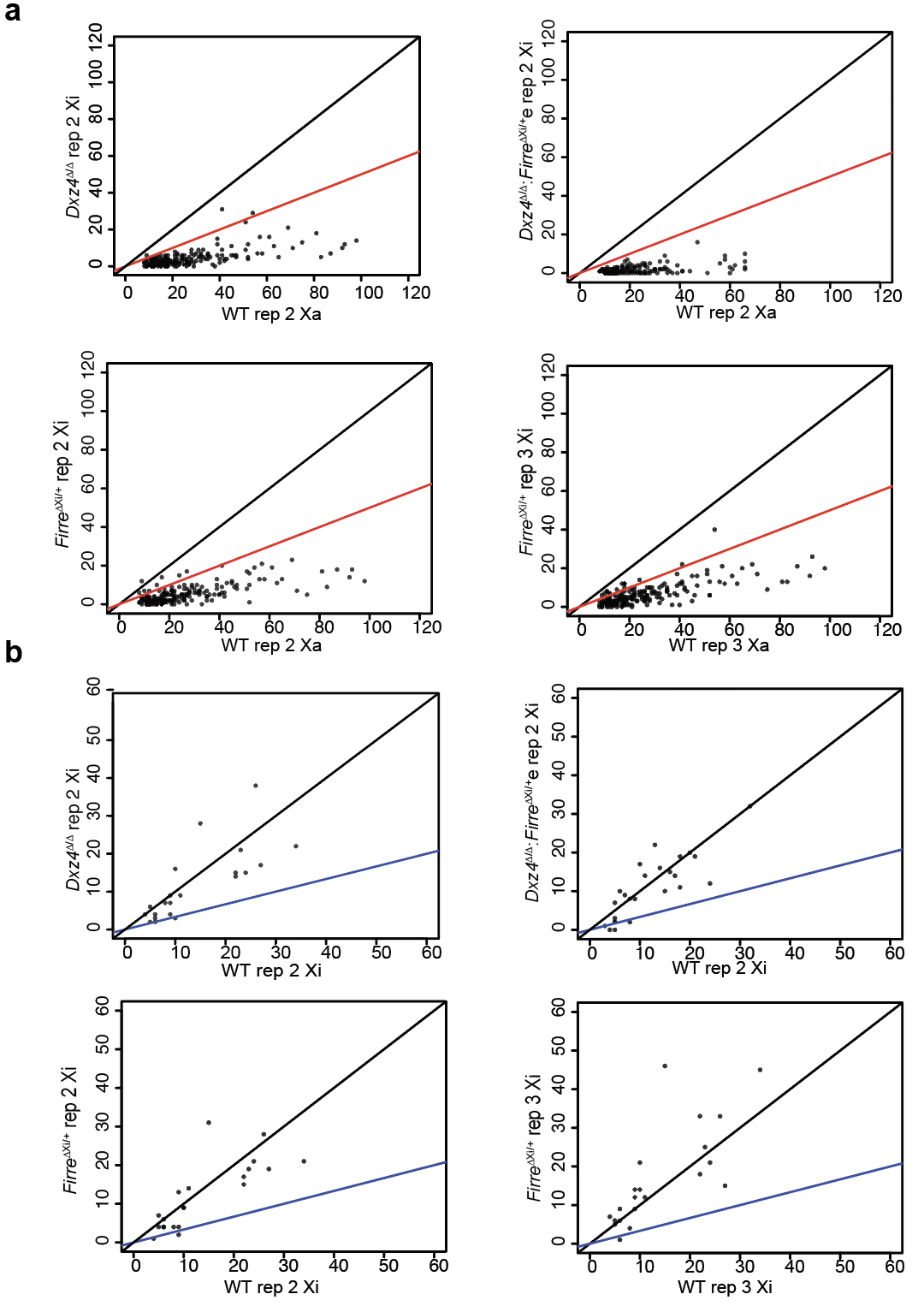
Supplementary Figure 10: Replicate ATAC-seq analysis in *Firre*Xi^∆/+^ and *Dxz4*^∆/∆^:*Firre*^Xi∆/+^.**

**a** Comparison between WT Xa and mutant Xi for ATAC coverage for peaks that are Xa-specific in wild-type for replicate ATAC-seqs. Top left: *Dxz4*^∆/∆^ Xi vs WT Xa, top right: *Dxz4*^∆/∆^:*Firre*^Xi∆/+^ Xi vs WT Xa, Bottom left: *Firre*^Xi∆/+^ Xi vs WT Xa, replicate 2, Bottom left: Firre^Xi∆/+^ Xi vs WT Xa, replicate 3. Black lines corresponds to a *Dxz4*^∆/∆^ Xi:WT Xa ratio of 1:1, red lines corresponds to a ratio of 1:2. **b** Comparison between WT Xa and mutant Xi ATAC coverage for peaks that are Xi-specific in wild-type for replicate ATAC-seqs. Top left: *Dxz4*^∆/∆^ Xi vs WT Xi, top right: *Dxz4*^∆/∆^:*FirreXi*^∆/+^ Xi vs WT Xi, Bottom left: *Firre*^Xi∆/+^ Xi vs WT Xi, replicate 2, Bottom left: *Firre*^Xi∆/+^ Xi vs WT Xi, replicate 3. ­Black lines correspond to a *Dxz4*^∆/∆^ Xi:WT Xi ratio of 1:1, blue lines correspond to a ratio of 1:2.

**Supplementary Table 1:** guide RNAs used in this study.

| Name | Sequence | mm9 Coordinates | Target |
| --- | --- | --- | --- |
| Firre_mus_right | TCCTAGAGACGCCCTcACGG | chrX:47988639-47988658 | Firre, 129/mus allele, upstream of Firre promoter |
| Firre_mus_left | AAACACTAGCACGGACgCTC | chrX:47903516-47903531 | Firre, 129/mus allele, downstream of Firre 3' UTR |
| Firre_cas_right | TCCTAGAGACGCCCTgACGG | chrX:47988639-47988658 | Firre, 129/mus allele, upstream of Firre promoter |
| Firre_cas_left | AAACACTAGCACGGACaCTC | chrX:47903516-47903531 | Firre, 129/mus allele, downstream of Firre 3' UTR |
| Dxz4∆100_left | GGTGGGTGCTTTTACCCACG | chrX:72919855-72919874 | Dxz4, adjacent to large CTCF peak DS-TR |
| Dxz4∆100_right | GCTAGCTCCACTCCTATTGGT | chrX:73024482-73024501 | Dxz4, upstream of Dxz4 |

**Note:** Lowercase letters represent allele-specific positions of *Firre* gRNAs.

**Supplementary Table 2: DNA FISH probes**

| **Target** | **Type** | **Source** | **BAC or fosmid ID** | **mm9 coordinates** |
| --- | --- | --- | --- | --- |
| Dxz4 | fosmid | CHORI | WIBR1-0428I12 | chrX:72972712-73013287 |
| Firre | fosmid | CHORI | WIBR1-0755K22 | chrX:47917649-47961442 |
| Stc1 | BAC | CHORI | RP24-340H12 | chr14:69487669-69694814 |
| Xist | plasmid | Lee Lab (Erwin et al., 2012, PMID: 22942124) | Sx9, available from Lee Lab | chrX:100679268-100650410 |
| Tg backbone | plasmid | Lee Lab (Chu et al., 2017, PMID: 28692038) | P1-puromycin plasmid, available from Lee Lab | NA (derived against P1 backbone) |

**Supplementary Table 3:** 4C oligonucleotides used in this study.

|  |  | **4C viewpoint primers** |  |  |
| --- | --- | --- | --- | --- |
| Name | Viewpoint | Sequence | 1st enzyme | 2nd enzyme |
| p706-Dpn2 | Dxz4 core | /5Biosg/CCT ACA CGA CGC TCT TCC GAT CTT GGG TGG AGT GCA GTG TTG AGA CA | DpnII | FatI |
| p711-Dpn2 | Sphinx core | /5Biosg/CCT ACA CGA CGC TCT TCC GAT CTC TKC TCC TCA GCC ATT TTG AAG C | DpnII | FatI |
| p710-Dpn2 | Sphinx 3' | /5Biosg/CCT ACA CGA CGC TCT TCC GAT CTC AGA GGC CAA TGT GTA GGC | DpnII | FatI |
| Hygro_4C_DpnIIprimer | Hygro marker in Xist Tg backbone | /5Biosg/ccctacacgacgctcttccgatctNNNNGAGCAGCAGACGCGCTACTT | DpnII | FatI |
|  |  | **4C adaptors and primers for ligation-mediated PCR** |  |  |
| Name | Description | Sequence |  |  |
| p637-FatI6N-TSad2 | adaptor top strand for FatI-digested libraries | /5Phos/CAT GNN NNN NAG ATC GGA AGA GCA CAC GTC TGA ACT C |  |  |
| p640-rc6N-TSad2 | adaptor bottom strand for 4C adaptors | GCT CTT CCG ATC TNN NNN N/3Phos/ |  |  |
| p521-TruSeq2rc | Library primer for ligation-mediated PCR; to be used with the biotinylated viewpoint primer | GAG TTC AGA CGT GTG CTC TTC CGA TCT |  |  |

**Supplementary Table 4:** qRT-PCR primers used in this study.

| Name | Amplicon | Sequence |
| --- | --- | --- |
| JR_Firre1_F | JR1 | GGAGAAAGGCAGAAATGCAG |
| JR_Firre1_R | JR1 | CAGTGTTCCAGCTCCAGTGA |
| JR_Firre2_F | JR2 | AGGTATGCTTCACCTCTCCT |
| JR_Firre2_R | JR2 | CAAATTCAAGCAGGCAAGGG |
| JR_Firre4_F | JR4 | TTTTTCATGCAGGGTGATTG |
| JR_Firre4_R | JR4 | AACAGTGCCCATTTCAGTCC |
| Disteche_Firre_F | CD | ACCAGGTACCGTGAGCAATC |
| Disteche_Firre_R | CD | TTCCTCATTCCCCTTCTCCT |
| Firre_int_set1_F | Firre_intronic | CCTGCCTACACATGCTACAA |
| Firre_int_set1_R | Firre_intronic | CAGGTCTTTGGGTCTTCCTATC |
| Xist_ex1-3_F | Xist_exon1-3 | GCTGGTTCGTCTATCTTGTGGG |
| Xist_ex1-3_R | Xist_exon1-3 | CAGAGTAGCGAGGACTTGAAGAG |
| Gapdh_F | Gapdh | ATGAATACGGCTACAGCAACAGG |
| Gapdh_R | Gapdh | GAGATGCTCAGTGTTGGGGG |
