# Supplementary material for: Megadomains and superloops form dynamically but are dispensable for X-chromosome inactivation and gene escape
Source: Nat Commun. 2018 Nov 27;9:5004. doi: 10.1038/s41467-018-07446-w (PMC6258728; doi:10.1038/s41467-018-07446-w)
Supplement: Supplementary file 2 — Reporting Summary [file 41467_2018_7446_MOESM2_ESM.pdf]

nature research

Corresponding author(s): *Double-blind peer review submissions: write DBPR and your manuscript number here instead of author names.*

# Reporting Summary

Nature Research wishes to improve the reproducibility of the work that we publish. This form provides structure for consistency and transparency in reporting. For further information on Nature Research policies, see Authors & Referees and the Editorial Policy Checklist.

## Statistical parameters

When statistical analyses are reported, confirm that the following items are present in the relevant location (e.g. figure legend, table legend, main text, or Methods section).

| n/a | Confirmed | |
|---|---|---|
| ☐ | ☒ | The exact sample size (*n*) for each experimental group/condition, given as a discrete number and unit of measurement |
| ☐ | ☒ | An indication of whether measurements were taken from distinct samples or whether the same sample was measured repeatedly |
| ☐ | ☒ | The statistical test(s) used AND whether they are one- or two-sided<br>*Only common tests should be described solely by name; describe more complex techniques in the Methods section.* |
| ☒ | ☐ | A description of all covariates tested |
| ☐ | ☒ | A description of any assumptions or corrections, such as tests of normality and adjustment for multiple comparisons |
| ☐ | ☒ | A full description of the statistics including central tendency (e.g. means) or other basic estimates (e.g. regression coefficient) AND variation (e.g. standard deviation) or associated estimates of uncertainty (e.g. confidence intervals) |
| ☐ | ☒ | For null hypothesis testing, the test statistic (e.g. *F*, *t*, *r*) with confidence intervals, effect sizes, degrees of freedom and *P* value noted<br>*Give P values as exact values whenever suitable.* |
| ☒ | ☐ | For Bayesian analysis, information on the choice of priors and Markov chain Monte Carlo settings |
| ☒ | ☐ | For hierarchical and complex designs, identification of the appropriate level for tests and full reporting of outcomes |
| ☒ | ☐ | Estimates of effect sizes (e.g. Cohen's *d*, Pearson's *r*), indicating how they were calculated |
| ☐ | ☒ | Clearly defined error bars<br>*State explicitly what error bars represent (e.g. SD, SE, CI)* |

*Our web collection on statistics for biologists may be useful.*

## Software and code

Policy information about availability of computer code

| | |
|---|---|
| Data collection | No software was used. |
| Data analysis | sequence alignment: cutadapt v1.7.1, novoalign_v3-1.00.02, bowtie2 v2.1.0 bedtools2 v2.25.0, samtools v0.1.18, homer/4.8, juicebox v7.5. Custom scripts to perform allele-specific analysis, generate and analyze Hi-C, data using homer, juicebox and R. |

For manuscripts utilizing custom algorithms or software that are central to the research but not yet described in published literature, software must be made available to editors/reviewers upon request. We strongly encourage code deposition in a community repository (e.g. GitHub). See the Nature Research guidelines for submitting code & software for further information.

## Data

Policy information about availability of data

All manuscripts must include a data availability statement. This statement should provide the following information, where applicable:

- Accession codes, unique identifiers, or web links for publicly available datasets
- A list of figures that have associated raw data
- A description of any restrictions on data availability

All sequencing data has been deposited in the GEO Accession GSE116649 [https://www.ncbi.nlm.nih.gov/geo/query/acc.cgi?acc=GSE116649]

¬All other relevant data are available from J. Froberg or J. Lee upon request.

# Field-specific reporting

Please select the best fit for your research. If you are not sure, read the appropriate sections before making your selection.

☒ Life sciences ☐ Behavioural & social sciences ☐ Ecological, evolutionary & environmental sciences

For a reference copy of the document with all sections, see nature.com/authors/policies/ReportingSummary-flat.pdf

# Life sciences study design

All studies must disclose on these points even when the disclosure is negative.

| | |
|---|---|
| Sample size | No sample size calculation performed because none is needed. |
| Data exclusions | No data exclusions. |
| Replication | All replicates are reported. |
| Randomization | Randomization not needed. |
| Blinding | Blinding was not needed for any experiments. |

# Reporting for specific materials, systems and methods

## Materials & experimental systems

| n/a | Involved in the study |
|---|---|
| ☒ | ☐ Unique biological materials |
| ☐ | ☒ Antibodies |
| ☐ | ☒ Eukaryotic cell lines |
| ☒ | ☐ Palaeontology |
| ☒ | ☐ Animals and other organisms |
| ☒ | ☐ Human research participants |

## Methods

| n/a | Involved in the study |
|---|---|
| ☒ | ☐ ChIP-seq |
| ☒ | ☐ Flow cytometry |
| ☒ | ☐ MRI-based neuroimaging |

## Antibodies

| | |
|---|---|
| Antibodies used | H3K27me3: Active Motif 39155, nucleophosmin: abcam 10530 |
| Validation | Antibodies were validated based on the validation information provided by the manufacturer. Both antibodies were used for immunoflouresence experiments, and both antibodies successfully labeled the expected nuclear structures (the Barr body for AM39155, the nucleolus for abcam 10530), and these images are included in the manuscript. |

## Eukaryotic cell lines

Policy information about cell lines

| | |
|---|---|
| Cell line source(s) | The following cell lines were used in these experiments: TsixTST/+ is a female mESC line, derived in the Lee lab and described in Ogawa et al., 2008. The male XY rtTA fibroblast line was derived in the Lee lab and described in Jeon et al., 2011. All additional cell lines used are derivatives of one of these cell lines generated in the Lee Lab. |
| Authentication | The genotypes of the relevant cell lines were confirmed in our high-throughput sequencing experiments. All deletion cell lines derived from TsixTST were validated by PCR spanning the deletion as well as DNA FISH using probes internal to the deletion. |
| Mycoplasma contamination | Mycoplasma contamination not assessed. |
| Commonly misidentified lines (See ICLAC register) | *Name any commonly misidentified cell lines used in the study and provide a rationale for their use.* |
